# Real Sample Consistency Regularization for GANs

**DOI:** 10.3390/e23091231

**Published:** 2021-09-19

**Authors:** Xiangde Zhang, Jian Zhang

**Affiliations:** College of Sciences, Northeastern University, Shenyang 110819, China; 1900138@stu.neu.edu.cn

**Keywords:** generative adversarial networks, zero gradient penalty, real sample consistency regularization, mode collapse

## Abstract

Mode collapse has always been a fundamental problem in generative adversarial networks. The recently proposed Zero Gradient Penalty (0GP) regularization can alleviate the mode collapse, but it will exacerbate a discriminator’s misjudgment problem, that is the discriminator judges that some generated samples are more real than real samples. In actual training, the discriminator will direct the generated samples to point to samples with higher discriminator outputs. The serious misjudgment problem of the discriminator will cause the generator to generate unnatural images and reduce the quality of the generation. This paper proposes Real Sample Consistency (RSC) regularization. In the training process, we randomly divided the samples into two parts and minimized the loss of the discriminator’s outputs corresponding to these two parts, forcing the discriminator to output the same value for all real samples. We analyzed the effectiveness of our method. The experimental results showed that our method can alleviate the discriminator’s misjudgment and perform better with a more stable training process than 0GP regularization. Our real sample consistency regularization improved the FID score for the conditional generation of Fake-As-Real GAN (FARGAN) from 14.28 to 9.8 on CIFAR-10. Our RSC regularization improved the FID score from 23.42 to 17.14 on CIFAR-100 and from 53.79 to 46.92 on ImageNet2012. Our RSC regularization improved the average distance between the generated and real samples from 0.028 to 0.025 on synthetic data. The loss of the generator and discriminator in standard GAN with our regularization was close to the theoretical loss and kept stable during the training process.

## 1. Introduction

Since the generative adversarial network proposed by Goodfellow [1] in 2014, it has achieved great development [2,3] and has been applied in many ways [4,5,6,7,8,9], such as image inpainting, super-resolution reconstruction, style transfer, and image editing. However, researchers are still looking for ways to improve GANs, especially ways to solve the mode collapse and instability of GANs [10,11,12]. Thanh-Tung [13] argued that the generated samples and the real samples in the later training stage are very similar, but the discriminator can distinguish between the real samples and the generated samples, resulting in a gradient explosion. In this case, the generator’s gradient in the minibatch points to samples where the gradient explodes and mode collapse occurs.

Thanh-Tung proved that when the generated distribution approaches the real distribution, the generator’s gradient should tend to zero. Therefore, the author proposed 0GP regularization on the linear interpolation between the real samples and the generated samples to alleviate mode collapse. Mescheder [14] proved that 0GP regularization on real samples could guarantee convergence when initialized sufficiently close to equilibrium.

However, experiments in this paper showed that both 0GP regularizations on the linear interpolation between real samples and generated samples and 0GP regularization on real samples exacerbated the discriminator’s misjudgment, that is the discriminator output has a higher value for generated samples than real samples. The discriminator’s misjudgment makes it more difficult for the generated samples to converge to the real samples and guides the generator to generate unnatural images, reducing the quality of the generation.

It is necessary for the discriminator to output higher values for some generated samples than the real samples, because if the discriminator can perfectly distinguish the generated samples from the real samples, this will cause the training to collapse. However, if there are massive generated samples for which the discriminator outputs higher values, this will reduce the quality of the generation. In the actual training, the discriminator will direct the generated samples to point to samples with higher discriminator outputs, regardless of whether they are real samples. If the discriminator judges that the massive generated samples are more real than the generated samples, then the generator’s gradient within a minibatch will be more directed toward the generated samples with a high discriminator output rather than the real samples. The result is that the generator generates many meaningless images, reducing the quality of the generation. 0GP regularization will exacerbate the problem of the discriminator’s misjudgment.

Tao and Wang [15] proposed fake-as-real GAN based on 0GP regularization on real samples. When updating the discriminator, the generated samples with the lowest discriminator output in the minibatch should be regarded as real samples. However, the problem of the discriminator’s misjudgment is still unresolved.

This paper focuses on solving the discriminator’s misjudgment and achieving better performance with a more stable training process. Our contributions are as follows:

1. We analyze the discriminator’s misjudgment. Due to the 0GP regularization, there will be more cases where the discriminator’s gradient at the real samples is less than the discriminator’s gradient at the generated samples during the training process;
2. We propose Real Sample Consistency (RSC) regularization, forcing the discriminator to output the same value for all real samples. For real samples, real sample consistency regularization can reduce the proportion of the discriminator output to be less than $\frac{1}{2}$. Experiments on synthetic and real-world datasets verified that our method achieves better performance than 0GP regularization.

## 2. Related Work

Researchers have been committed to improving generative adversarial networks. Reference [16] utilized the Wasserstein distance and the clip parameter to regularize GANs. The Wasserstein distance can solve the vanishing gradient problem, but the clip parameters will decrease the model’s fitting ability. Reference [17] proposed One Gradient Penalty (1GP) regularization, which improves the fitting ability of the model, but does not guarantee model convergence. Mescheder [14] proved that 0GP regularization on real samples could guarantee convergence when initialized sufficiently close to equilibrium. Reference [10] proposed spectral normalization to make the model have Lipschitz continuity to stabilize the training process. Reference [18] proposed consistency regularization, which makes the model insensitive to data augmentation and can maintain consistency in the semantic feature space, thereby improving the model’s performance. Reference [15] proposed that the generated samples with the lowest discriminator output in the minibatch should be regarded as real samples, thus achieving better generalization.

Reference [19] proposed to replace the sigmoid cross-entropy loss in the standard GAN with the mean-squared error loss to solve the vanishing gradient problem. Hinge loss [20] and ResNet [21] are also applied to GANs to improve the performance. Reference [22] utilized instance noise to alleviate the vanishing gradient problem. Reference [23] utilized the Exponential Moving Average (EMA) to update the generator, which can stabilize the training of the process. Reference [24] argued that only considering the current generator when updating the generator will lead to mode collapse, so the author proposed that the current generator be considered, and the discriminator after *K* iterations should be considered. References [25,26,27] utilized multiple generators to alleviate model collapse. References [11,12,28] achieved amazing results and could generate realistic high-resolution pictures.

## 3. Approach

In this part, we focus on the problem of the discriminator’s misjudgment. We analyze the problem of misjudgment by the discriminator and propose the real sample consistency regularization.

### 3.1. Background

The discriminator of the Standard GAN (SGAN) proposed in 2014 maximizes:

(1) $$L=E_{x∼p_{r}}\left[\log\left(D\left(x\right)\right)\right]+E_{y∼p_{g}}[\log\left(1-D\left(y\right)\right],$$

where $p_{r}$ represents the real distribution and $p_{g}$ represents the generated distribution. Reference [1] proposed that when the generator is fixed, the optimal discriminator is:

(2) $$D^{*}\left(v\right)=\frac{p_{r}\left(v\right)}{p_{r}\left(v\right)+p_{g}\left(v\right)},\forall v\in \mathrm{supp}\left(p_{r}\right)\cup \mathrm{supp}\left(p_{g}\right)$$

When the global optimum is reached, there are: $p_{r}=p_{g}$ and $D^{*}\left(v\right)=\frac{1}{2}$. Reference [15] mentioned that for any $v\in \mathrm{supp}\left(p_{r}\right)\;\cup \;\mathrm{supp}\left(p_{g}\right)$, when $p_{g}$ approaches $p_{r}$, $D^{*}\left(v\right)$ approaches $\frac{1}{2}$, and ${\left(\nabla D\right)}_{v}\to 0$, thereby ${\left(\nabla D\right)}_{x}\to 0$, $x\in p_{r}$.

Therefore, there are two ways to perform 0GP regularization. One enforces a zero-centered gradient penalty of the form ${∥{\left(\nabla D\right)}_{x}∥}^{2}$, where $x∼p_{r}$. The discriminator maximizes:

(3) $$\begin{array}{c} L_{0GP-R}=E_{x∼p_{r}}\left[\log\left(D\left(x\right)\right)\right]+E_{y∼p_{g}}[\log\left(1-D\left(y\right)\right]\\ -\mu E_{x∼p_{r}}{∥{\left(\nabla D\right)}_{x}∥}^{2}, \end{array}$$

where μ>0. The other enforces a zero-centered gradient penalty of the form ${∥{\left(\nabla D\right)}_{v}∥}^{2}$, where *v* is a linear interpolation between real samples and generated samples. The discriminator maximizes:

(4) $$\begin{array}{c} L_{0GP-I}=E_{x∼p_{r}}\left[\log\left(D\left(x\right)\right)\right]+E_{y∼p_{g}}[\log\left(1-D\left(y\right)\right]\\ -\mu E{∥{\left(\nabla D\right)}_{v}∥}^{2}, \end{array}$$

where μ>0.

### 3.2. Misjudgment by the Discriminator

However, no matter what the form of zero-centered gradient penalty, it is far from perfect regularization.

**Definition** **1.** *For $y_{0}\in S_{g}$, $y_{0}$ is a fake real sample if $y_{0}\in \left\{y_{0}:D\left(y_{0}\right)>E\left[D\left(S_{r}\right)\right]\right\}$. $S_{r}$ represents the set of real samples. $S_{g}$ represents the set of generated samples.*

Consider the 0GP regularization on real samples. Although the 0GP regularization of the real sample can alleviate the mode collapse, it will lead to D(y)>D(x), which indicates that the discriminator believes that some generated samples are more real than the real samples. We can infer that SGAN-0GP will show more fake real samples than SGAN. Since ${\left(\nabla D\right)}_{x}\to 0$ only occurs near the equilibrium point and we apply 0GP regularization from the beginning of the training, this will lead the gradient of the discriminator at the real samples to be close to 0. Moreover, we did not impose restrictions on the gradient of the discriminator at the generated samples. As a result, there will be more cases where the discriminator’s gradient at the real samples is less than the discriminator’s gradient at the generated samples during the training process. In the end, the number of fake real samples in SGAN-0GP will be more than that in SGAN. The empirical discriminator guides the generated samples to point to samples with higher discriminator outputs, regardless of whether they are real samples. Therefore, the discriminator will guide the generator to generate more fake real samples. These fake real samples are often far from the real samples and eventually lead to difficulty in convergence.

0GP regularization on the linear interpolation between real and generated samples leads to D(y)>D(x) as well. Although, in this case, the zero-centered gradient penalty is applied on the linear interpolation between real and generated samples, the number of real samples is finite, and the number of generated samples is infinite. Assume that $S_{r}$ represents the set of real samples, $S_{g}$ represents the set of generated samples, and Reg represents the total quantity of the regularizations. Then, $∥S_{r}∥\ll ∥S_{g}∥$, $\frac{Reg}{∥S_{g}∥}\ll \frac{Reg}{∥S_{r}∥}$. The penalty for the gradient of the discriminator at the generated samples is much less than that at the real samples. Therefore, we can infer that the number of fake real samples generated by SGAN with 0GP on real samples is similar to that on the linear interpolation between real and generated samples.

Considering 0GP regularization on the linear interpolation between real and generated samples is more complicated than that on real samples, and the result of interpolation may not lie in $\mathrm{supp}\left(p_{r}\right)\;\cup \;\mathrm{supp}\left(p_{g}\right)$ [15]. In the rest of the paper, we use the 0GP regularization on real samples by default.

### 3.3. Real Sample Consistency Regularization

In order to alleviate the problem of fake real samples, we can increase $D(S_{r})$ and decrease $D(y_{0})$. Assume that for $x_{c}\in S_{r}$, $\left\{x_{c},y_{i}\right\}$ is a close pair for ∀$y_{i}\in \left\{y_{1},\cdots ,y_{n}\right\},n>1$. According to the definition of a close pair, we can approximate $y_{i}\in \left\{y_{1},\cdots ,y_{n}\right\}$ as $x_{c}$, because according to the previous assumption of n>1, we can obtain $p_{g}\left(x_{c}\right)>p_{r}\left(x_{c}\right)$. Consider Equation (2); we have $D^{*}\left(x_{c}\right)<\frac{1}{2}$. This shows that although $x_{c}$ is a real sample, the discriminator’s output for $x_{c}$ is less than $\frac{1}{2}$. Therefore, we propose Real Sample Consistency (RSC) regularization, which enforces the discriminator to output the same value for the real samples. Considering that the proportion of the discriminator’s output for real samples <$\frac{1}{2}$ is low and >$\frac{1}{2}$ is high, this alleviates the problem of the discriminator’s output for real samples $<\frac{1}{2}$ by enforcing the discriminator to output the same value for the real samples. The discriminator in SGAN-RSC maximizes:

(5) $$\begin{array}{cc} \; & L_{rsc}=E_{x∼p_{r}}\left[\log\left(D\left(x\right)\right)\right]+E_{y∼p_{g}}[\log\left(1-D\left(y\right)\right]-\\ & \mu E_{x∼p_{r}}{∥{\left(\nabla D\right)}_{x}∥}^{2}-\lambda E_{x_{s},x_{t}∼p_{r}}{∥D\left(x_{s}\right)-D\left(x_{t}\right)∥}^{2}, \end{array}$$

where μ>0,λ>0. The training procedure is presented in Algorithm 1.

**Algorithm 1:** Minibatch stochastic gradient descent training
of SGAN-RSC.

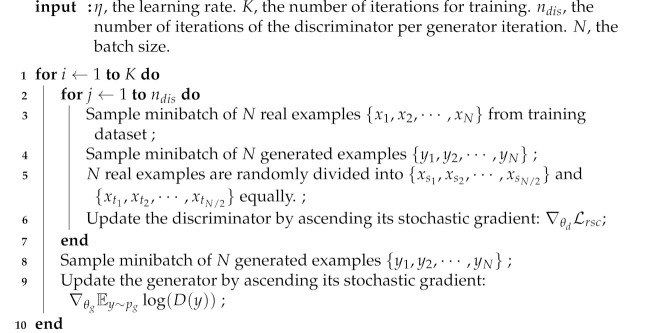

**Definition** **2.** *For $x_{c}\in S_{r}$, $y_{c}\in S_{g}$, {$x_{c},y_{c}$} is a δ close pair if $y_{c}\in \left\{y_{c}:d\left(x_{c},y_{c}\right)\leq \delta ,0<\delta \ll d\left(x_{i},x_{j}\right),\forall x_{i},x_{j}\in S_{r}\right\}$ [15]. $S_{r}$ represents the set of real samples, and $S_{g}$ represents the set of generated samples.*

Assume that for $x_{c}\in S_{r}$, $x_{c}$ does not belong to any close pair, then the discriminator will output a high value for $x_{c}$. Our proposed RSC regularization can alleviate this problem. Regularization on real samples will also affect the generated samples due to the adversarial learning.

## 4. Experimental Results

To verify the effectiveness of our proposed real sample consistency regularization, we experimented on synthetic data, CIFAR-10, CIFAR-100, and ImageNet2012. The optimizers of all our experiments were set to RMSProp, α = 0.99, ϵ = 1 × 10^−8^. The learning rates of the generator and the discriminator were both set to 1 × 10^−4^. The batch size was set to 64. Once the discriminator was updated, the generator was updated once. To achieve better results, instance noise [22] and the exponential moving average [23] were applied, and the β of the exponential moving average was set to 0.999. In order to verify the effectiveness of our method on different network architectures and different datasets, we applied ResNet [21] and traditional network architectures [29] on CIFAR-10 and CIFAR-100. The network architecture of ResNet was the same as [13], and the traditional network architecture was the same as [11]. We did not use batch normalization. The FID score [30] was selected to evaluate the generated samples, and a lower FID value represents better generation. The FID value was obtained on 10 k generated samples. We used Pytorch for development.

### 4.1. Synthetic Data

Sample *N* examples $\left\{x_{1},x_{2},\cdots ,x_{N}\right\}$ from two-dimensional normal distribution $N\left(0,0;1,1;0\right)$, denoted as $X_{sample}$. For each training, $X_{sample}$ was fixed. The synthetic data can be obtained by $X_{\mathrm{synthesis}\;}=X_{\mathrm{sample}\;}+\psi Z$, where ψ is 0.02, $Z∼N\left(0,0;1,1;0\right)$. In our experiment, there were three settings for *N*, namely 25, 50, and 100, which are denoted as 25 Gaussians, 50 Gaussians, and 100 Gaussians, respectively. Considering the dimension of the synthetic data to be two, we used MLP as the network architecture; see Table A1 and Table A2 in the Appendix A for the details. In this dataset, we set μ=200,λ=500.

**Figure 1:**
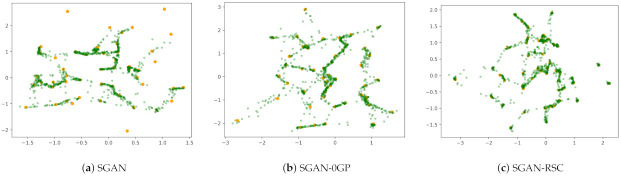
Result of SGAN, SGAN-0GP, and SGAN-RSC on 50 Gaussians at 100 k iterations. SGAN, SAGN-0GP, and SGAN-RSC were iterated 100 k times on 50 Gaussians. The orange dots represent real samples, and the green dots represent randomly generated samples. There are 1000 green points in each subfigure.

We verified our previous analysis by the experiments on the synthetic dataset. As shown in Figure 1, SGAN-0GP showed more fake real samples during the training process than SGAN. The number of fake real samples generated by SGAN with 0GP on real samples was similar to that on the linear interpolation between real and generated samples. We can also find that 0GP regularization on real samples and 0GP regularization on the linear interpolation between real and generated samples resulted in the number of fake real samples exceeding that in SGAN. These observations are consistent with our previous analysis in Section 3.

**Figure 2:**
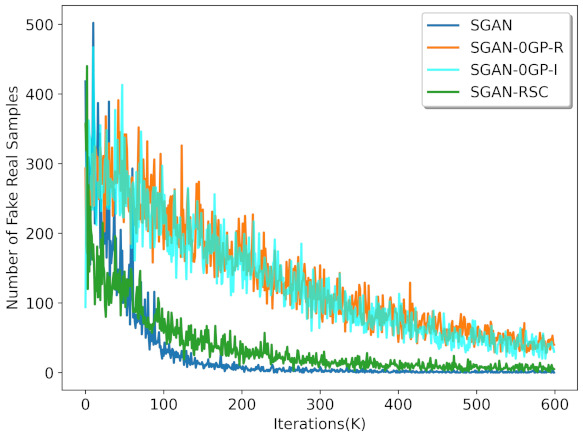
Result of the number of fake real samples in SGAN, SGAN-0GP, and SGAN-RSC with ResNet on CIFAR-10. We randomly sampled 1000 fake samples and counted the number of false samples. SGAN-0GP-R represents 0GP regularization on real samples; SGAN-0GP-I represents 0GP regularization on the linear interpolation between the real samples and the generated samples.

Figure 1 shows the qualitative results of SGAN, SGAN-0GP, and SGAN-RSC on the synthetic data. The ideal generation is to generate samples that cover every real sample and that every generated sample be close to the real samples. However, we note that the green generated samples did not cover part of the orange real samples in Figure 1a, which indicates that mode collapse occurred. In Figure 1b, although mode collapse did not occur, many generated samples were distributed far away from the real samples. The generation in Figure 1c was the best in the three subfigures. Not only was there no mode collapse, but there were fewer generated samples that were far away from the real samples. The consequence was consistent with Figure 2. Although the number of fake real samples in SGAN was low, mode collapse occurred. According to Figure 1 and Figure 2, the SGAN-RSC we proposed led to fewer fake real samples and generated samples that were far away from the real samples and avoided model collapse.

The quantitative results were consistent with the qualitative results in Figure 1. As shown in Table 1, the average distance obtained by our method was less than the average distance obtained by 0GP, and as the number of real samples increased, the better the improvement of our method. Our method’s improvement for 25 Gaussians was less than that for 50 Gaussians and 100 Gaussians because the 25 Gaussian dataset was simple, and the average distance was close to the ideal average distance.

### 4.2. CIFAR-10 and CIFAR-100

In CIFAR-10 and CIFAR-100, we set the image resolution at 32 × 32 and experimented on both the conventional network and ResNet. We set μ = 10, λ = 20 for the conventional network architecture and μ = 10, λ = 500 for ResNet, see Table A3, Table A4, Table A5 and Table A6 in the Appendix A for the details. The results are shown in Table 2.

**Figure 3:**
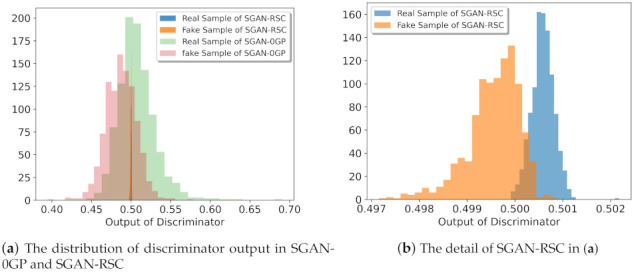
Result of the output of discriminator in SGAN-0GP and SGAN-RSC with ResNet on CIFAR-10. We randomly sampled 1000 real samples and 1000 fake samples. The statistical discriminator’s output distribution for real samples and fake samples, respectively.

We verified our previous analysis in Section 3 by experiments on CIFAR-10 and CIFAR-100. As shown in Figure 3, the discriminator’s output with our regularization for both real samples and fake samples was more concentrated. For real samples, the proportion of the discriminator with our regularization output less than $\frac{1}{2}$ was lower than the proportion of the discriminator with 0GP regularization output less than $\frac{1}{2}$. For fake samples, the proportion of the discriminator with our regularization output greater than $\frac{1}{2}$ was lower than the proportion of the discriminator with 0GP regularization output greater than $\frac{1}{2}$. This was consistent with the result in Figure 2. As shown in Figure 2, the number of fake real samples in SGAN-RSC was lower than the number of fake real samples in SGAN-0GP.

**Figure 4:**
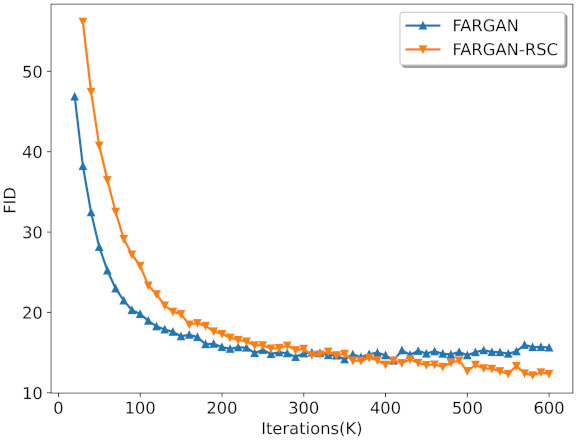
Result on FARGAN and FARGAN-RSC on CIFAR-10 with the conventional network architecture.

The result on FARGAN and FARGAN-RSC on CIFAR-10 is shown in Figure 4. FARGAN-RSC outperformed FARGAN. Note that the FID of FARGAN dropped quickly at the beginning of training. However, as the training progressed, the FID of FARGAN was surpassed by the FID of FARGAN-RSC, and the FID of FARGAN-RSC continued to decrease.

**Figure 5:**
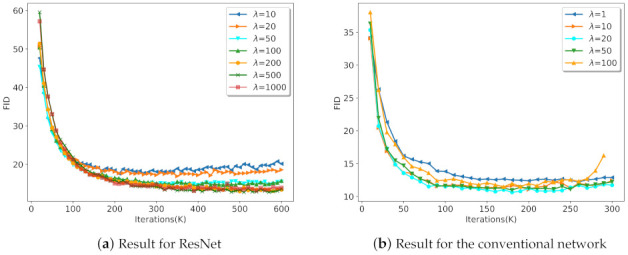
Result for SGAN-RSC with different λ on CIFAR-10 with the conventional network and ResNet.

In order to obtain the optimal parameter λ, we selected parameter λ through ablation experiments, as shown in Figure 5. Figure 5a shows that, when λ<500, SGAN-RSC with ResNet achieved better results with the increase of λ. However, when we set λ=1000, the result was worse than that of λ=500. Consequently, we set λ=500 for SGAN-RSC with ResNet. Similarly, we set λ=20 for SGAN-RSC with the conventional network.

**Figure 6:**
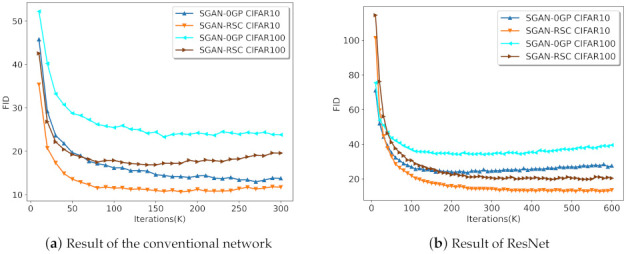
FID of SGAN-0GP and SGAN-RSC. Calculated on CIFAR-10 and CIFAR-100 with the conventional network and ResNet.

To verify the effectiveness of our proposed real sample consistency regularization with different network architectures, we compared SGAN-0GP and SGAN-RSC with the conventional network and ResNet, respectively. Experiments were carried out on CIFAR-10 and CIFAR-100. The result is shown in Figure 6. In all experiments, SGAN-RSC outperformed SGAN-0GP, especially with ResNet. Note that although the FID value of SGAN-RSC with the conventional architecture on CIFAR-100 increased slowly in the late training period, and the lowest FID value of SGAN-RSC with the conventional architecture on CIFAR-100 was lower than that of SGAN-0GP with the conventional architecture on CIFAR-100. Figure 6 shows that our method was effective for different network architectures.

**Figure 7:**
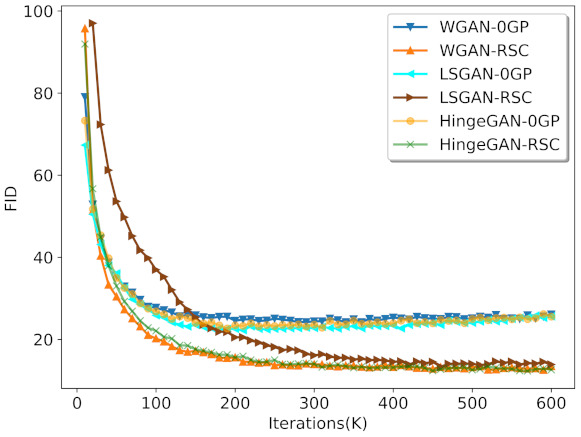
Result of 0GP regularization and RSC regularization on different GAN variants.

We also experimented with different GAN variants. In our experiments, 0GP on real samples instead of 1GP was applied in WGAN [16,17,31]. We set a=c=1,b=0 for LSGAN [19] and N=64,M=32,f=8, and $N_{0}=16$ for FARGAN [15]. The result is shown in Figure 7. Note that real sample consistency regularization outperformed 0GP regularization for all GAN variants. Although LSGAN-RSC converged slowly in the early stages of training, it eventually reached an FID value similar to that obtained by other GAN variants with real sample consistency regularization. Figure 7 shows that our method was effective for different GAN variants.

**Figure 8:**
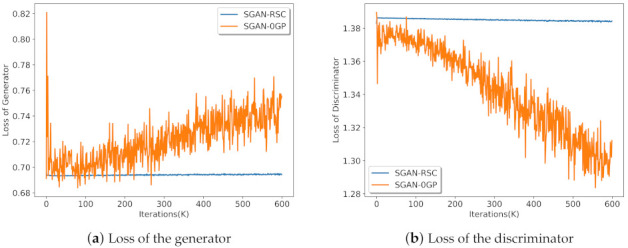
The loss of the generator and discriminator in SGAN-0GP and SGAN-RSC with ResNet on CIFAR-10.

The loss of the generator and discriminator with ResNet on CIFAR-10 is shown in Figure 8. The theoretical loss was ln2≈0.693 for the generator and 2ln2≈1.386 for the discriminator. As the training progressed, we observed that the loss of the generator increased, and the loss of discriminator decreased significantly in SGAN-0GP. However, the loss of the generator and discriminator in SGAN-RSC was close to the theoretical loss and kept stable. Figure 8 shows that our method can stabilize the training of SGAN-0GP.

**Figure 9:**
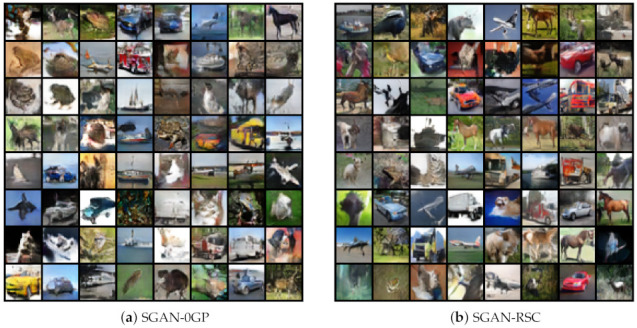
Randomly images generated by SGAN-0GP and SGAN-RSC with ResNet on CIFAR-10.

**Figure 10:**
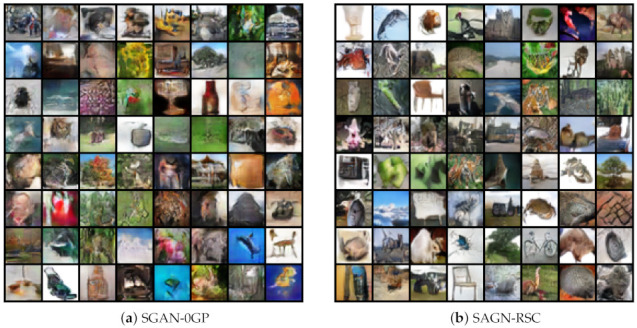
Randomly images generated by SGAN-0GP and SGAN-RSC with ResNet on CIFAR-100.

**Figure A1:**
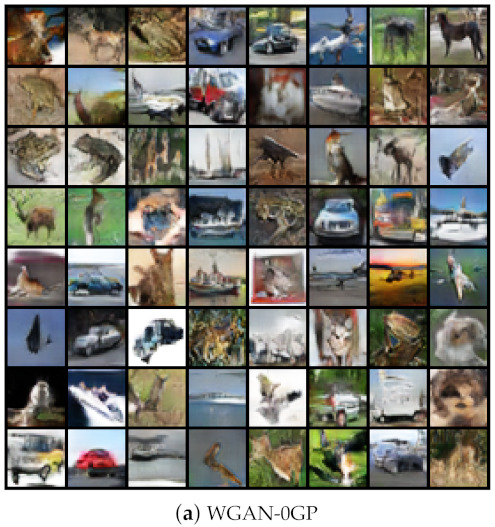

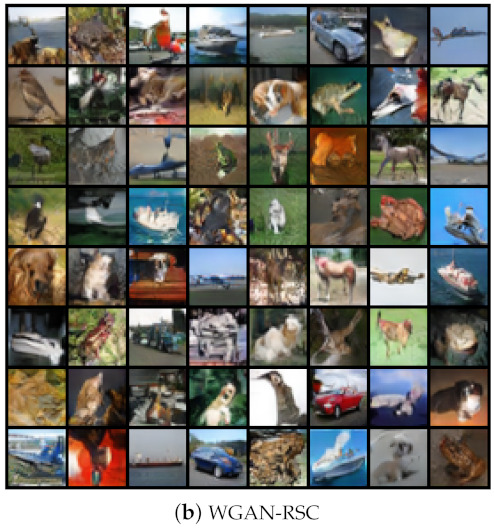
Randomly images generated by WGAN-0GP and WGAN-RSC with ResNet on CIFAR-10.

**Figure A2:**
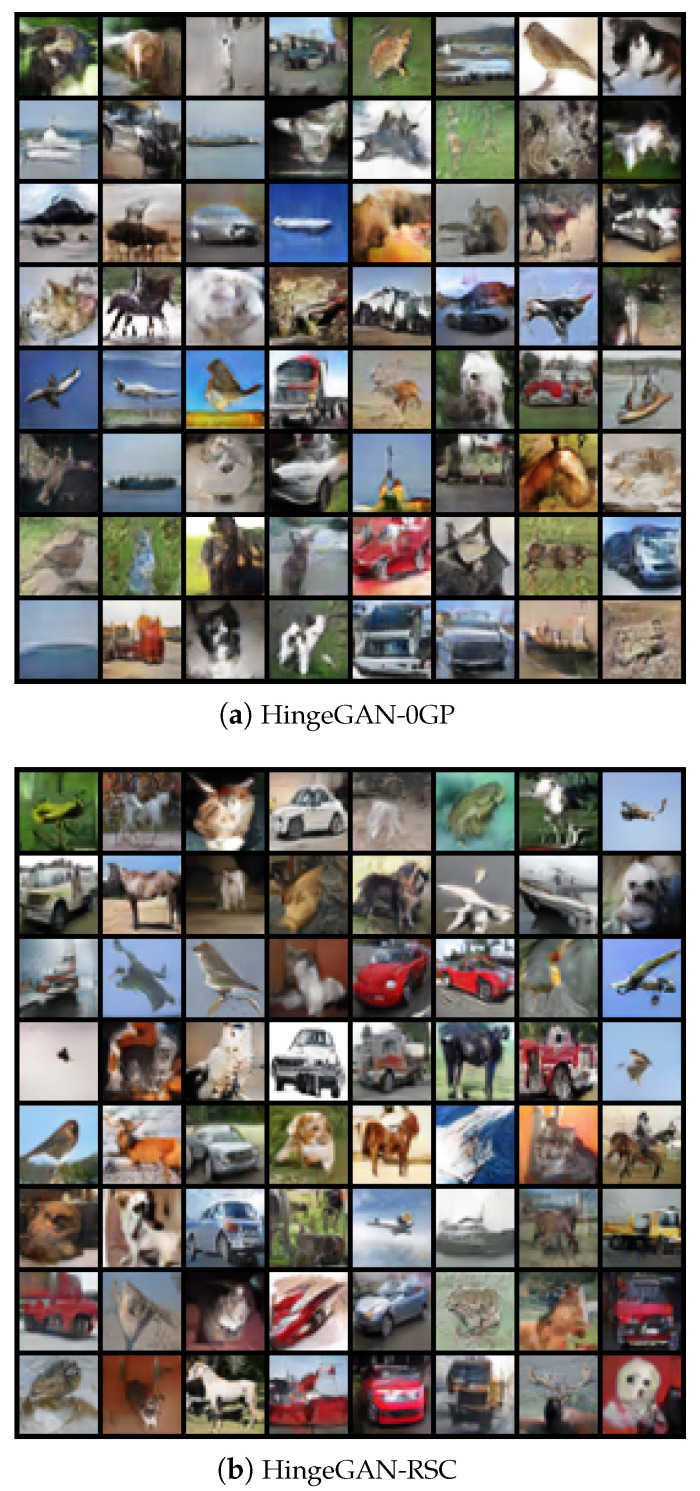
Randomly images generated by HingeGAN-0GP and HingeGAN-RSC with ResNet on CIFAR-10.

**Figure A3:**
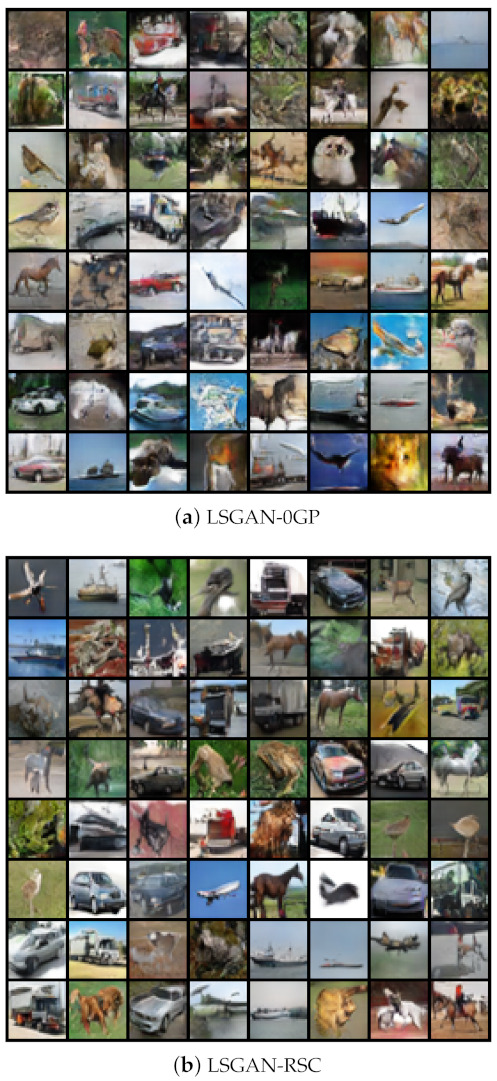
Randomly images generated by LSGAN-0GP and LSGAN-RSC with ResNet on CIFAR-10.

**Figure A4:**
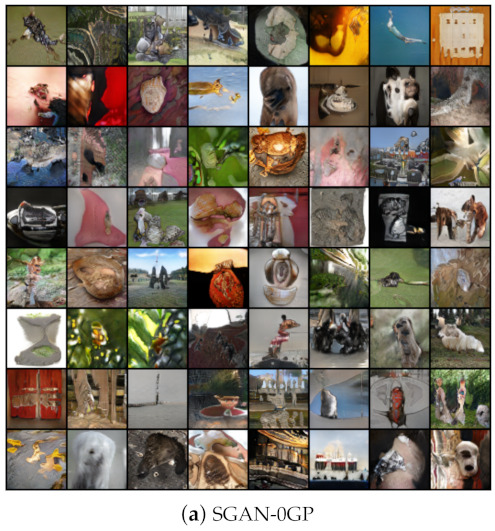

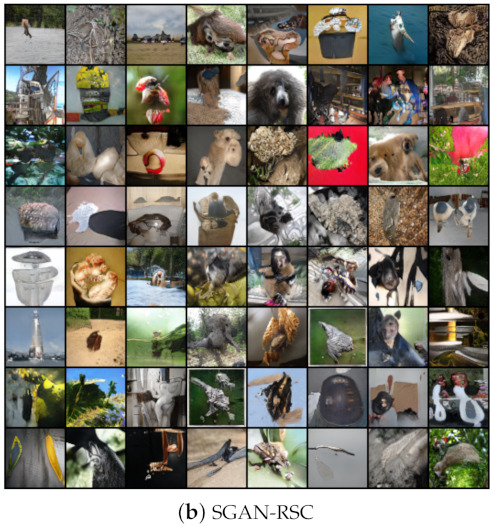
Randomly images generated by SGAN-0GP and SGAN-RSC with ResNet on ImageNet.

The qualitative result with ResNet on CIFAR-10 is shown in Figure 9. Note that the images generated by SGAN-RSC were sharper and the structure clearer, such as the images of horses, airplanes, and cars. SGAN-0GP generated more unnatural and fuzzy images due to the fake real samples. Figure 9 and Figure 10 show that our proposed regularization outperformed 0GP regularization qualitatively on CIFAR-10 and CIFAR-100. More qualitative results are shown in Figure A1, Figure A2, Figure A3 and Figure A4.

**Figure 11:**
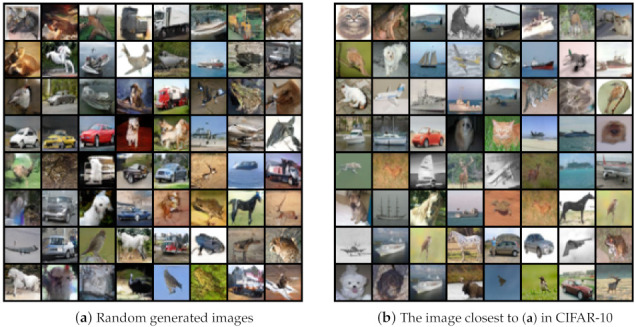
Randomly images generated by SGAN-RSC and the image closest to it in CIFAR-10. We used the cosine distance as the basis for comparison.

In Figure 11, we show the images randomly generated by SGAN-RSC and the images closest to the generated images in CIFAR-10. The results showed that our model did not copy the images, but learned the distribution of the real images, which can guarantee the diversity of the generation.

We compared other state-of-the-art methods, and the results are shown in Table 3. Real sample consistency regularization improved the FID score of FARGAN on CIFAR-10 from 14.28 to 9.8.

### 4.3. ImageNet

In order to verify the effectiveness of our method on challenging datasets, we experimented on ImageNet, which contains 1000 classes. We set the image resolution at 64 × 64 and experimented on ResNet; see Table A7 and Table A8 in the Appendix A for details. We set μ = 10, λ = 20. The results are shown in Table 4.

### 4.4. Summary of the Experimental Results

As shown in Table 5, the results of RSC regularization on all datasets surpassed the results of 0GP regularization. This shows that our method works for different datasets.

## 5. Conclusions

This paper showed that 0GP regularization introduces the discriminator’s misjudgment, which is the discriminator outputting a higher value for some generated samples than the real samples. We analyzed the discriminator’s output for the real sample, which was that a close pair with several generated samples was less than $\frac{1}{2}$. We proposed a new regularization, which forced the discriminator to output the same value for all real samples. The experiment result showed that our proposed regularization can reduce the number of fake real samples. Experiments on synthetic data showed that our method reduces the distance between the real distribution and the generated distribution and avoids mode collapse. Experiments on CIFAR-10, CIFAR-100, and ImageNet verified that our method can stabilize the training process and significantly improve the performance.

## Figures and Tables

**Table 1:** The average distance between the generated sample and the real samples on 25 Gaussians, 50 Gaussians, and 100 Gaussians at 100 k iterations. Experiments were repeated 10 times.

	SGAN-0GP	SGAN-RSC
25 Gaussians	0.028 ± 0.0053	0.025 ± 0.0048
50 Gaussians	0.058 ± 0.0084	0.048 ± 0.0102
100 Gaussians	0.072 ± 0.0094	0.057 ± 0.014

**Table 2:** FID on CIAFR-10 and CIFAR-100 with ResNet and the conventional network. Experiments were repeated 3 times.

	0GP	RSC
CIFAR-10		
ResNet SGAN	24.15 ± 0.27	12.05 ± 0.50
ResNet WGAN	24.33 ± 0.16	12.90 ± 0.07
ResNet LSGAN	22.32 ± 0.05	14.40 ± 0.40
ResNet HingeGAN	23.39 ± 0.12	12.37 ± 0.31
ResNet FARGAN	14.28 ± 0.16	11.66 ± 0.09
Conventional SGAN	13.12 ± 0.41	10.92 ± 0.04
CIFAR-100		
ResNet SGAN	34.48 ± 0.02	19.80 ± 0.19
Conventional SGAN	23.42 ± 0.29	17.14 ± 0.07

**Table 3:** Comparison with state-of-the-art GAN models including SNGAN [10], BigGAN [6], CR-BigGAN [18], and FARGAN [15]. The FID value of FARGAN-RSC was obtained at 1200 k iterations.

Method	SNGAN	BigGAN	CR-BigGAN	FARGAN	FARGAN-RSC (Ours)
FID	17.5	14.73	11.48	14.28	9.8

**Table 4:** FID of SGAN-0GP and SGAN-RSC on ImageNet with ResNet.

Method	SGAN-0GP	SGAN-RSC
FID	53.79	46.92

**Table 5:** The best results of 0GP and RSC on different datasets. On the synthetic data, we recorded the average distance between the generated sample and the real samples for comparison. On CIFAR-10, CIFAR-100, and ImageNet2012, we recorded the FID for comparison.

	0GP	RSC
Synthetic data (distance)	0.028	0.025
CIFARA-10 (FID)	13.12	9.8
CIFAR-100 (FID)	23.42	17.14
ImageNet2012 (FID)	53.79	46.92

## Data Availability

Not applicable.

## Appendix A

**Table A1:** Generator architecture in the synthetic experiment.

Layer	Output Size	Filter
Fully connected	64	2 → 64
RELU	64	–
Fully connected	64	64 → 64
RELU	64	–
Fully connected	64	64 → 64
RELU	64	–
Fully connected	2	64 → 2

**Table A2:** Discriminator architecture in the synthetic experiment.

Layer	Output Size	Filter
Fully connected	64	2 → 64
RELU	64	–
Fully connected	64	64 → 64
RELU	64	–
Fully connected	64	64 → 64
RELU	64	–
Fully connected	1	64 → 1

**Table A3:** Generator ResNet architecture in the CIFAR experiment.

Layer	Output Size	Filter
Fully connected	512 × 4 × 4	128 → 512 × 4 × 4
Reshape	512 × 4 × 4	–
ResNet-Block	256 × 4 × 4	512 → 256 → 256
NN-Upsampling	256 × 8 × 8	–
ResNet-Block	128 × 8 × 8	256 → 128 → 128
NN-Upsampling	128 × 16 × 16	–
ResNet-Block	64 × 16 × 16	128 → 64 → 64
NN-Upsampling	64 × 32 × 32	–
ResNet-Block	64 × 32 × 32	64 → 64 → 64
Conv2D	3 × 32 × 32	64 → 3

**Table A4:** Discriminator ResNet architecture in the CIFAR experiment.

Layer	Output Size	Filter
Conv2D	64 × 32 × 32	3→ 64
ResNet-Block	128 × 32 × 32	64 → 64 → 64
Avg-Pool2D	128 × 16 × 16	–
ResNet-Block	256 × 16 × 16	128 → 128 → 256
Avg-Pool2D	256 × 8 × 8	–
ResNet-Block	512 × 8 × 8	256 → 256 → 512
Avg-Pool2D	512 × 4 × 4	–
Reshape	512 × 4 × 4	–
Conv2D	1	512 × 4 × 4 → 1

**Table A5:** Generator conventional architecture in the CIFAR experiment.

Layer	Output Size	Filter
Fully connected	512 × 4 × 4	128 → 512 × 4 × 4
Reshape	512 × 4 × 4	–
Conv2D	512 × 4 × 4	512 → 512
Conv2D	512 × 4 × 4	512 → 512
NN-Upsampling	512 × 8 × 8	–
Conv2D	256 × 8 × 8	512 →256
Conv2D	256 × 8 × 8	256 → 256
NN-Upsampling	512 × 16 × 16	–
Conv2D	128 × 16 × 16	256 →128
Conv2D	128 × 16 × 16	128 → 128
NN-Upsampling	128 × 32 × 32	–
Conv2D	64 × 32 × 32	128 →64
Conv2D	64 × 32 × 32	64 → 64
Conv2D	3 × 32 × 32	64 → 3

**Table A6:** Discriminator conventional architecture in the CIFAR experiment.

Layer	Output Size	Filter
Fully connected	512 × 4 × 4	128 → 512 × 4 × 4
Reshape	512 × 4 × 4	–
Conv2D	64 × 32 × 32	3 → 64
Conv2D	64 × 32 × 32	64 → 64
Conv2D	128 × 32 × 32	64 → 128
Avg-Pool2D	128 × 16 × 16	–
Conv2D	128 × 16 × 16	128 → 128
Conv2D	256 × 16 × 16	128 → 256
Avg-Pool2D	256 × 8 × 8	–
Conv2D	256 × 8 × 8	256 → 256
Conv2D	512 × 8 × 8	256 → 512
Avg-Pool2D	512 × 4 × 4	–
Reshape	512 × 4 × 4	–
Fully connected	1	512 × 4 × 4→ 1

**Table A7:** Generator architecture in the ImageNet experiment.

Layer	Output Size	Filter
Fully connected	1024 × 4 × 4	256 → 1024 × 4 × 4
Reshape	1024 × 4 × 4	–
ResNet-Block	1024 × 4 × 4	1024 → 1024 → 1024
ResNet-Block	1024 × 4 × 4	1024 → 1024 →1024
NN-Upsampling	1024 × 8 × 8	–
ResNet-Block	512 × 8 × 8	1024 → 512 → 512
ResNet-Block	512 × 8 × 8	512 → 512 →512
NN-Upsampling	512 × 16 × 16	–
ResNet-Block	256 × 16 × 16	512 → 256 → 256
ResNet-Block	256 × 16 × 16	256 → 256 →256
NN-Upsampling	256 × 32 × 32	–
ResNet-Block	128 × 32 × 32	256 → 128 → 128
ResNet-Block	128 × 32 × 32	128 → 128 →128
NN-Upsampling	128 × 64 × 64	–
ResNet-Block	64 × 64 × 64	128 → 64 → 64
ResNet-Block	64 × 64 × 64	64 → 64 →64
Conv2D	3 × 64 × 64	64 → 3

**Table A8:** Discriminator architecture in the ImageNet experiment.

Layer	Output Size	Filter
Conv2D	64 × 64 × 64	3 → 64
ResNet-Block	64 × 64 × 64	64 → 64 → 64
ResNet-Block	128 × 64 × 64	64 → 64 →128
Avg-Pool2D	128 × 32 × 32	–
ResNet-Block	128 × 32 × 32	128 → 128 → 128
ResNet-Block	256 × 32 × 32	128 → 128 →256
Avg-Pool2D	256 × 16 × 16	–
ResNet-Block	256 × 16 × 16	256 → 256 → 256
ResNet-Block	512 × 16 × 16	256 → 256 →512
NN-Upsampling	512 × 8 × 8	–
ResNet-Block	512 × 8 × 8	512 → 512 → 512
ResNet-Block	1024 × 8 × 8	512 → 512 →1024
NN-Upsampling	512 × 4 × 4	–
ResNet-Block	1024 × 4 × 4	1024 → 1024 →1024
ResNet-Block	1024 × 4 × 4	1024 → 1024 →1024
Fully connected	1	1024 × 4 × 4→ 1
